# In silico chemical screening identifies epidermal growth factor receptor as a therapeutic target of drug-tolerant CD44v9-positive gastric cancer cells

**DOI:** 10.1038/s41416-019-0600-9

**Published:** 2019-10-14

**Authors:** Tetsuo Mashima, Risa Iwasaki, Naomi Kawata, Ryuhei Kawakami, Koshi Kumagai, Toshiro Migita, Takeshi Sano, Kensei Yamaguchi, Hiroyuki Seimiya

**Affiliations:** 1 Division of Molecular Biotherapy, Cancer Chemotherapy Center, Japanese Foundation for Cancer Research, Tokyo, Japan; 2 Department of Computational Biology and Medical Sciences, Graduate School of Frontier Sciences, The University of Tokyo, Tokyo, Japan; 3 Gastroenterological Medicine, Cancer Institute Hospital, Japanese Foundation for Cancer Research, Tokyo, Japan; 4 Gastroenterological Surgery, Cancer Institute Hospital, Japanese Foundation for Cancer Research, Tokyo, Japan

**Keywords:** Cancer stem cells, Cancer therapeutic resistance

## Abstract

**Background:**

Tumours consist of heterogeneous cancer cells and are likely to contain drug-tolerant cell subpopulations, causing early relapse. However, treatment strategies to eliminate these cells have not been established.

**Methods:**

We established gastric cancer patient-derived cells (PDCs) to examine the contribution of CD44 splicing variant 9 (CD44v9)-positive cells in gastric cancer drug tolerance. We performed gene expression signature-based in silico screening using JFCR_LinCAGE, our anticancer compound gene expression database and subsequent validation in BALB/c-nu/nu mouse xenograft to identify agents targeting the drug-tolerant cancer cells.

**Results:**

CD44v9-positive cancer cells were enriched among residual cancer cells after treatment with SN-38, an active metabolic of irinotecan. CD44v9 protein was responsible for this drug resistance. We identified epidermal growth factor receptor (EGFR) inhibitors as agents that can target CD44v9-positive cell populations in gastric cancer PDCs. CD44v9 promoted cell proliferation, and EGFR inhibition attenuated CD44v9 protein expression through downregulation of the AKT and the ERK signalling pathways, leading to preferential suppression of CD44v9-positive cells. Importantly, EGFR inhibitors significantly reduced the number of residual cancer cells after cytotoxic anticancer drug treatment and enhanced the antitumor effect of irinotecan in vivo.

**Conclusions:**

EGFR inhibitors could be potential agents to eradicate cytotoxic anticancer drug-tolerant gastric cancer cell populations.

## Background

Gastric cancer is a common cancer worldwide and is a major cause of cancer-related death in Asian countries.^1^ For the treatment of patients with metastatic, recurrent, or advanced gastric cancer, conventional chemotherapy based on 5-fluorouracil (5-FU), irinotecan, or other drugs, and molecularly targeted agents, such as ramucirumab and trastuzumab, are used as standard therapy.^2^ However, therapeutic effects are largely limited by intrinsic or acquired drug resistance and early relapse after treatment.^3^

Accumulating evidence has indicated that tumour tissues consist of heterogenous cancer cells^4^ and contain subpopulations of intrinsically drug-resistant cancer cells even before treatment, which may remain as ‘persister cells’ after treatment and hamper its effectiveness.^5,6^ Particularly, cancer stem cells, defined as a specific subset of cancer cells with highly tumorigenic and drug-resistant potential, are believed to contribute to the early phases of drug resistance.^7,8^ In gastric cancer, a population of cancer cells expressing splicing variants of a cell surface transmembrane glycoprotein, CD44, (CD44v), particularly CD44v9 containing variant exons 8–10, was shown to possess cancer stem cell properties.^9,10^ Moreover, CD44v9 expression was significantly associated with poor prognosis in gastric cancer.^11^ These observations suggest that this subset of gastric cancer cells could be responsible for the early phases of drug resistance, though no direct validation study of this notion in a gastric cancer patient-derived cell model has yet been performed.

CD44v9 contributes to cell proliferation and survival by reducing anti-proliferative reactive oxygen species (ROS) levels through interacting with and regulating xCT, a cystine transporter.^9^ Sulfasalazine, a drug developed to treat inflammatory bowel disease and rheumatoid arthritis, was identified as a xCT-selective inhibitor. Clinical trials of sulfasalazine were performed in gastric cancer for targeting CD44v9-positive cancer stem cells,^12^ but faced some problems in dose escalation due to the emergence of adverse effects (anorexia). Thus, there is still no approved drug that targets the drug-resistant subpopulations of CD44v9-positive cancer cells.

In silico drug screening approaches based on accumulated databases of cancer cell drug sensitivity and gene expression related to drug action are potentially attractive for identifying novel links between drugs and diseases, because they are less time-consuming or/and cost-consuming methods of obtaining new evidence for drug repositioning.^13–15^ Previously, we developed a public database of gene expression changes in cancer cells treated with approved antitumor agents and cancer-related intracellular signalling inhibitors, named JFCR_LinCAGE (Japanese Foundation for Cancer Research Database of Link between Chemotherapeutic Agents and Gene Expression).^16^ This database is useful for evaluating target molecular pathways of test compounds whose modes of action are not fully known.^17^ Moreover, the database could also be applied to identifying new agents that target malignant populations of cancer cells by comparing gene expression signatures in the database^18^ and further validating antitumor effect of candidate agents in vitro and in the mouse xenograft model.

In this study, we first confirmed that CD44v-positive cancer cells are persister cells after cytotoxic antitumor agent treatment in our newly developed patient-derived gastric cancer cell model. An in silico drug screening approach with our database successfully identified epidermal growth factor receptor (EGFR) inhibitors as agents that can target drug-tolerant CD44v-positive gastric cancer cells.

## Methods

### Establishment of patient-derived gastric cancer cell lines

Surgical specimens of gastric cancers were obtained at the Cancer Institute Hospital of Japanese Foundation for Cancer Research (Tokyo, Japan) under the approval of an institutional review board (IRB) with written informed consent of the patients.

Pieces of tumours from the patients were obtained after surgical resection and cut into small fragments, then washed with ice-cold phosphate-buffered saline (PBS) supplemented with antibiotic–antimycotic (Thermo Fisher Scientific, Waltham, MA). Then, the tumour pellets were digested with EZ enzyme (Kurabo, Osaka, Japan) for 2 h. After washing with antibiotic–antimycotic-containing PBS, the cell pellets were cultured in PCM-2 (Kurabo) or in ACL4 medium (Thermo Fisher Scientific) to establish patient-derived cell lines, JSC15-3, 17–2 and 17–7. Before the start of the experiments, the cells were sub-cultured until coexisting stromal cells were rarely detectable by microscopy.

Culture conditions of the cell lines used in this study are described in the Supplementary Materials and Methods.

### Chemical compounds

5-FU, SN-38 and temsirolimus were purchased from Sigma (St Louis, MO). Afatinib and trametinib were purchased from Selleck Chemicals (Houston, TX) or Funakoshi (Tokyo, Japan). Erlotinib was purchased from Cell Signaling Technology (Danvers, MA). Irinotecan (CAMPTO) was obtained from Yakult Honsha Co. Ltd (Tokyo, Japan). Cycloheximide was obtained from Nacalai Tesque (Kyoto, Japan).

### Cell proliferation and colony-formation assays

Cell proliferation was evaluated using thiazolyl blue tetrazolium bromide (MTT) (Sigma). Cells were seeded into 96-well microplates and treated with drugs. For the measurement of cell viability, MTT was added to the medium at a final concentration of 0.8 mg/mL. After incubation for 4 h, the medium containing MTT was removed and dimethyl sulfoxide (DMSO) was added. Optical density (OD) at 570 nm and 630 nm (reference) was measured. For colony-formation assay, cells were seeded into six-well plates and cultured for 14 days in the presence of DMSO (control) or indicated concentrations of drugs. Colonies were stained with 0.5% crystal violet/25% methanol and quantified by ImageJ software (National Institute of Health, Bethesda, MD) with normalisation to the control cells, which were defined as 100%.

### Flow cytometry and cell sorting

CD44v9-positive and CD44v9-negative cell populations were analysed by flow cytometry. Single-cell suspensions were incubated with rat anti-human CD44v9 antibody (Cosmobio, Tokyo, Japan) for 30 min at 4 °C, washed three times in suspension buffer (PBS containing 25 mmol/L HEPES (pH, 7.0), 1 mmol/L EDTA and 0.5% foetal bovine serum (FBS)), incubated further with phycoerythrin (PE)-conjugated anti-rat IgG (Life Technology, Carlsbad, CA) for 30 min at 4 °C, and then washed three times with suspension buffer. The antibody-stained cells were analysed with a FACScalibur flow cytometer (BD Biosciences, San Jose, CA). For cell sorting, cells were stained as described above, and cell sorting was performed using a FACSAria or FACSAriaIII cell sorter (BD Biosciences) in accordance with the manufacturer’s protocol.

### Immunohistochemistry

Tissue microarrays containing gastric cancer tissues were obtained from Funakoshi. Xenograft tumours were obtained as described below, and formalin-fixed, paraffin-embedded tissues were prepared. After deparaffinization and heat-induced epitope retrieval, as described previously,^19^ the sections were incubated with rat anti-CD44v9 antibody (Cosmobio) at 4 °C overnight. The ChemMate Envision Kit/horseradish peroxidase (Agilent Technologies (Dako), Santa Clara, CA) was used for detection and staining intensity was scored semi-quantitatively.

### Retrovirus-mediated stable gene expression

Retrovirus expression vector for CD44v9 was constructed as described in the Supplementary Materials and Methods. Retrovirus was produced as described previously^19^, and JSC15-3 CD44v9(−) cells were transduced with CD44v9 virus. Infected cells were selected by 300 µg/mL G418 to establish JSC15-3 CD44v9(−) cells stably overexpressing exogenous CD44v9.

### cDNA microarray and JFCR_LinCAGE analysis

The total RNA was extracted from JSC15-3 CD44v9(+) and CD44v9(−) cells using a RNeasy mini kit (Qiagen, Hilden, Germany). cDNA microarray analysis was performed with extracted RNA as described previously^16^ using the GeneChip Human Genome U133 Plus 2.0 Array (Thermo Fisher Scientific (Affymetrix)). Gene expression data were normalised using the RMA method and a JSC15-3 CD44v9(+) cell-specific signature gene set (genes whose expression was more than threefold higher or lower in CD44v9(+) cells than in CD44v9(–) cells) was further extracted with GeneSpring GX software (Agilent Technologies). The JFCR_LinCAGE analysis programme can compare ‘query’ gene signatures with drug-related gene signatures in the database (http://scads.jfcr.or.jp/db/cs/).^16^ Utilising this program, we extracted compounds in our database whose treatment reduced the expression of genes selectively expressed in the JSC15-3 CD44v9(+) cells, as described previously.^17^ The gene expression data have been deposited in Gene Expression Omnibus (GEO) and are accessible through the accession number GSE129747. The data will be released on March 31, 2020.

### Western blot analysis

Cells were lysed in whole-cell extract (WCE) lysis buffer (150 mM NaCl, 1.0% Nonidet P-40 (NP-40), 50 mM Tris-HCl, pH 8.0) supplemented with 1 × protease inhibitor cocktail (Nacalai) and PhosSTOP phosphatase inhibitor cocktail (Roche, Basel, Switzerland). Western blot analysis was performed as described previously.^19^ The antibodies used in this study are listed in the Supplementary Materials and Methods.

### Reverse transcription-quantitative PCR (RT-qPCR)

The total RNA was extracted using a RNeasy Mini kit (Qiagen). cDNA was synthesised using SuperScript III First-Strand Synthesis SuperMix for RT-qPCR (Life Technologies). RT-qPCR was performed using a LightCycler 96 (Roche). Because the CD44v8–10 form was the major CD44 splicing variant in our gastric cancer PDCs, we quantitated the mRNA levels of variant exon 10-containing *CD44*. Primer sequences for RT-qPCR were as follows: *CD44* forward primer (for variant exon 10): 5′-GGTGGAAGAAGAGACCCAAA-3′, *CD44* reverse primer: 5′-TTTGCTCCACCTTCTTGACTCC-3′, *ACTB* forward primer: 5′-ATTGGCAATGAGCGGTTC-3′, *ACTB* reverse primer: 5′-TGAAGGTAGTTTCGTGGATGC-3′.

### siRNA treatment

Silencer select siRNAs (targeting *EGFR*, *HER2* and *AKT1*) and Stealth siRNAs (targeting *CD44*) as well as negative control siRNAs were purchased from Thermo Fisher Scientific. siRNAs were introduced into cells using Lipofectamine RNAiMAX Transfection Reagent (Thermo Fisher Scientific). The siRNAs used in this study are listed in the Supplementary Materials and Methods.

### Therapeutic study in mouse xenograft models

All animal procedures were performed in the animal experiment room at JFCR using protocols approved by the JFCR Animal Care and Use Committee in compliance with the ARRIVE guideline. Experimental conditions and procedures, such as cancer cell implantation and the drug treatment schedule, are described in the Supplementary Materials and Methods.

## Results

### CD44v9-expressing cancer cells contribute to resistance to cytotoxic anticancer drugs in gastric cancer patient-derived cells (PDCs)

**Fig. 1:**
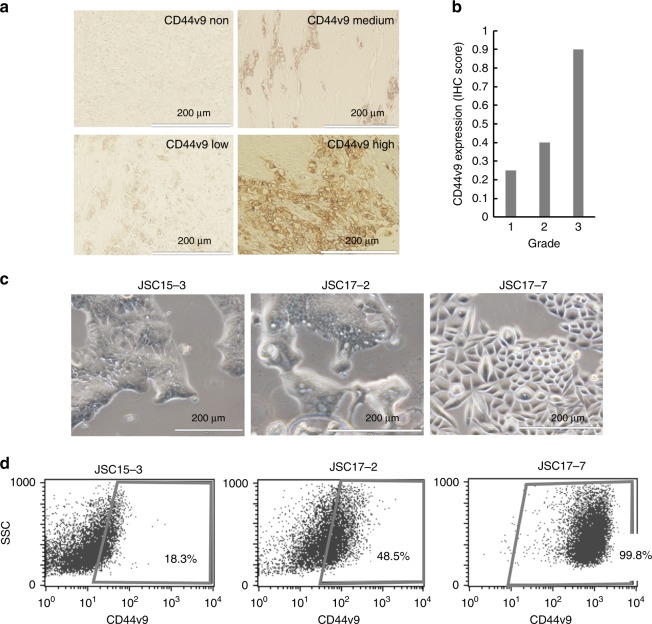
CD44 variant 9 (CD44v9) expression in gastric cancer patient-derived cells (PDCs). **a** CD44 variant 9 (CD44v9) expression in clinical gastric cancer tissues detected by immunohistochemistry (IHC). Expression levels were classified into four levels: non, low, medium and high. Typical staining patterns in each category are shown. **b** CD44v9 expression levels were scored as described in Supplementary Table 2. The average score in each grade is shown. **c** Morphologies of the established gastric cancer patient-derived cells (PDCs). **d** CD44v9-positive cancer cell populations in the gastric cancer PDCs as evaluated by flow-cytometry analysis

CD44v9-positive cancer cells with a malignant phenotype have been reported to exist as a subpopulation in gastric tumour tissues.^11^ To confirm this observation, we first conducted immunohistochemical studies on a gastric cancer tissue microarray. CD44v9-positive cancer cells were observed in 34.8% of gastric cancer tissues among 66 cases (typical tissues with high, medium and low/no CD44v9-positive cells are shown in Fig. 1a; the signal intensity in each sample is shown in Supplementary Table 1). CD44v9-positive cancer cells were frequently observed in higher grades of gastric cancer tissues (Fig. 1b; Supplementary Table 2), although the relationship between CD44v9 expression and grade was not statistically significant in χ-squared or Fisher’s exact tests. These data indicated heterogenous CD44v9 expression in gastric cancer tissues. To examine the involvement of the CD44v9-positive cell population in resistance to antitumor agents, we attempted to establish patient-derived cancer cells (PDCs) from the surgical specimens of gastric cancers obtained from March 2015 to May 2017 (Supplementary Table 3). We successfully established three cell lines, JSC15-3, JSC17-2 and JSC17-7 (Fig. 1c). As an initial characterisation of these cells, we examined expression status of differentiation-related markers and receptor tyrosine kinases (Supplementary Fig. 1A, B). We observed high MUC2 expression (a marker of intestinal type gastric cancer) in JSC15-3 cells and high MUC5AC and MUC6 expression (markers of stomach type gastric cancer) in JSC17-7 cells, which are consistent with differentiation status of original tumour tissues. On the other hand, JSC17-2 cells did not express either marker at high level, which raised a possibility that the original tumour could have contained complex types of cancer cells. The established PDCs contained differential proportions of CD44v9-positive cancer cells (Fig. 1d).

**Fig. 2:**
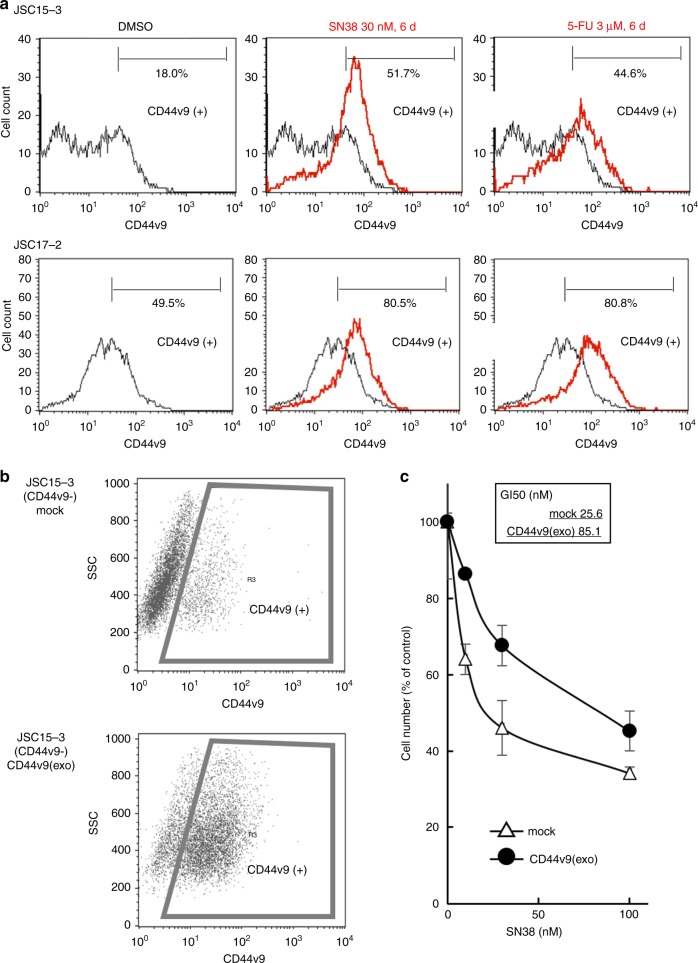
Involvement of CD44v9-positive cancer cells in resistance to cytotoxic antitumor agents in gastric cancer PDCs. **a** Accumulation of a CD44v9-positive cancer cell population among residual cancer cells after treatment with cytotoxic antitumor agents, SN-38 and 5-FU, in gastric cancer patient-derived cells. Cells were left untreated (DMSO) or treated with SN-38 (active metabolite of irinotecan) or 5-fluorouracil (5-FU) at the indicated concentrations for 6 days. CD44v9-positive cancer cell populations in untreated control cells (black) or in drug-treated cells (red) were evaluated by flow cytometry. **b** CD44v9 expression in CD44v9-negative JSC15-3 cells transduced with empty vector-derived virus (mock) or with CD44v9 retrovirus vector-derived virus (CD44v9(exo)) as estimated by flow cytometry. **c** Resistance of exogenous CD44v9-overexpressing JSC15-3 cells to SN-38. Cell numbers were measured by the MTT method as described in the Materials and Methods. Error bars indicate standard deviation

To examine the involvement of CD44v9-positive cells in resistance to antitumor agents, we first compared the number of CD44v9-positive cells before and after treatment with standard anticancer drugs for gastric cancer, such as SN-38 (an active metabolite of irinotecan) and 5-FU. We observed markedly enriched CD44v9-positive cancer cells among the persister cells in JSC15-3 and 17–2 cells with heterogenous CD44v9 expression (Fig. 2a), but not in JSC17-7 cells, which expressed ubiquitously high levels of CD44v9 (Supplementary Fig. 2A). In JSC15-3 cells, we further examined the drug sensitivity of the residual cancer cells after drug treatment. Persister cells enriched with CD44v9-positive cells showed resistance to SN-38 and 5-FU (Supplementary Fig. 3A). Alternative splicing of *CD44* results in the expression of various CD44 splicing variants. Among those variants, CD44v9 has been reported as a marker of gastric cancer stem cells.^9,10^ Consistent with these observations, cloning and sequencing of the *CD44v* cDNA isolated from JSC15–3 cells revealed that *CD44v9* was the major form expressed in these cells (Supplementary Fig. 3B). The *CD44v* splicing pattern as evaluated by PCR analysis using each variant-specific primer pair revealed no alteration in the splicing patterns before and after SN-38 or 5-FU treatment (Supplementary Fig. 4).

To further determine the involvement of CD44v9 in drug resistance, we genetically manipulated the *CD44v9* gene in gastric PDCs. We sorted the CD44v9-positive and CD44v9-negative cells from JSC15-3 cells (Supplementary Fig. 5A) and cloned the *CD44v9* cDNA from the CD44v9-positive cells. When we retrovirally transferred the *CD44v9* gene into the CD44v9-negative cells (Fig. 2b), the CD44v9-expressing cells acquired resistance (3.3-fold in GI50 value) to SN-38 (Fig. 2c), but not to 5-FU (data not shown).

These observations indicated that CD44v9-positive cells contribute to drug resistance as persister cells after drug treatment of gastric cancer. Moreover, CD44v9 directly contributed to SN-38 resistance.

To clarify the character of cancer stem cells in the CD44v9-expressing cells, we further examined the expression of cancer stem cell markers.^7^ We observed elevated expression of Sox2 and ABCG2, but not of CD24, in the CD44v9-positive cells (Supplementary Fig. 5B), while CD133 levels were low and could not be detected in CD44v9-positive and CD44v9-negative cells (data not shown). By contrast, the rate of spheroid growth, another character of cancer stem cells, was similar in the CD44v9-positive and CD44v9-negative cells (Supplementary Fig. 5C). These data suggest that the CD44v9-positive cells would possess a certain characters of cancer stem cells, such as drug resistance and stem cell-related gene expression in our gastric cancer PDC model.

### In silico chemical screening identified EGFR inhibitors as agents targeting SN-38- and 5-FU-tolerant gastric cancer CD44v9-expressing cells

**Fig. 3:**
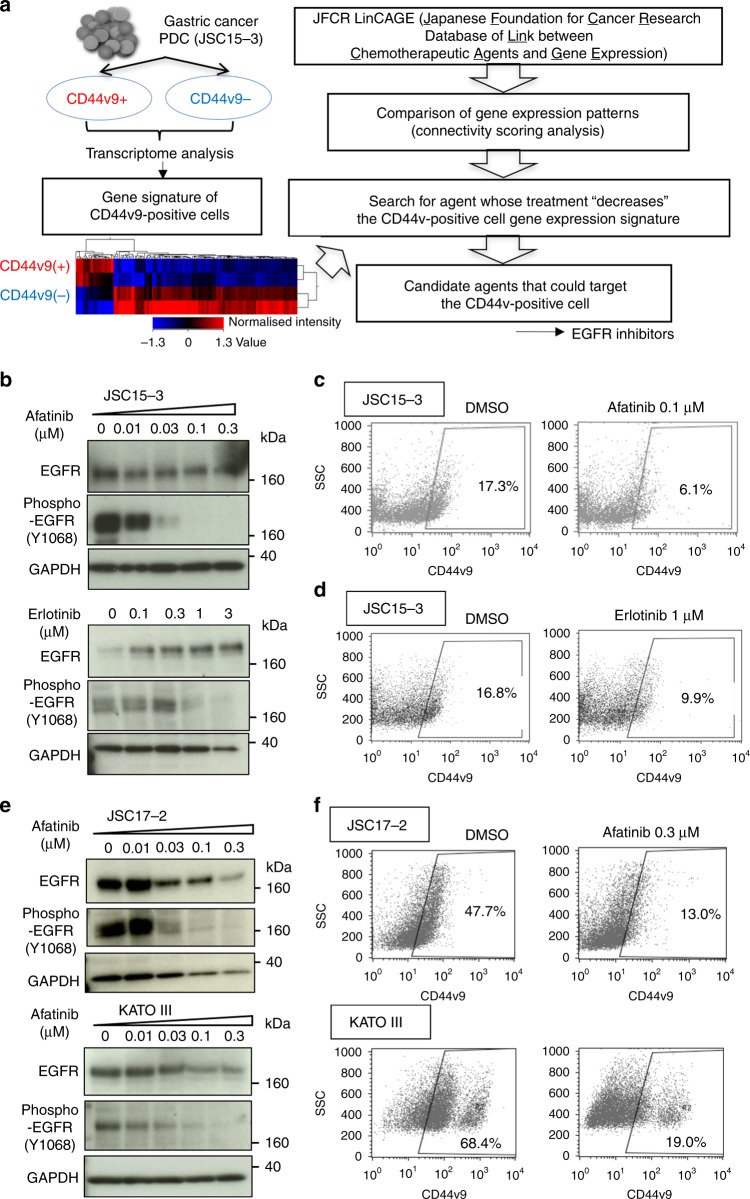
Gene expression-based in silico screening for agents targeting drug-tolerant CD44v9-positive gastric cancer cells. **a** Search strategy for candidate agents that target drug-tolerant CD44v9 cancer cells utilising gene expression data for CD44v9-positive and CD44v9-negative JSC15-3 cells in the JFCR_LinCAGE (Japanese Foundation for Cancer Research Database of Link between Chemotherapeutic Agents and Gene Expression) database (http://scads.jfcr.or.jp/db/cs/). **b**, **e** Effect of EGFR inhibitor treatment on EGFR autophosphorylation in gastric cancer cells. Cells were treated with afatinib or erlotinib at the indicated concentrations for 6 days. EGFR autophosphorylation at Tyr1068 and EGFR protein levels were examined by immunoblot analysis. **c**, **d**, **f** Elimination of CD44v9-positive cancer cell populations by EGFR inhibitor treatment in gastric cancer cells. Cells were left untreated (DMSO) or treated with afatinib or erlotinib at the indicated concentrations for 6 days. CD44v9-positive cancer cell populations were evaluated by flow cytometry

To identify agents that target CD44v9-positive cancer cells, we utilised a gene signature-based approach with our JFCR_LinCAGE database.^17^ For the analysis, we first performed transcriptome analysis using the human genome U133 plus 2.0 microarray on CD44v9-positive and CD44v9-negative cells sorted from JSC15-3 cells (Supplementary Fig. 5A). We next extracted genes that were more than threefold upregulated or downregulated in the CD44v9-positive cells as the CD44v9-positive cell signature gene set. Then, we extracted compounds in our database that suppress the expression of the CD44v9-positive cell signature gene set as candidate CD44v9-targeting agents based on connectivity scoring analysis^16^ (Fig. 3a). As a result, several EGFR inhibitors, including afatinib, erlotinib and gefitinib, emerged as candidate hit compounds (Supplementary Table 4).

To validate whether the EGFR inhibitors could act as CD44v9-positive cell-targeting agents, we treated the gastric PDCs with afatinib and erlotinib. As shown in Fig. 3b, when JSC15-3 cells were treated with afatinib or erlotinib, dose-dependent suppression of EGFR autophosphorylation was observed, and the CD44v9-positive cell population was markedly decreased (Fig. 3c, d). Of note, the dosages of afatinib required to decrease the CD44v9-positive cell population (0.03–0.3 µM) corresponded to its clinically applicable concentrations.^20^ Similar results were obtained in other gastric cancer PDCs, JSC17-2 cells, as well as in an established gastric cancer cell line, KATO III with heterogenous CD44v9 expression (Fig. 3e, f). By contrast, in JSC17-7 cells that ubiquitously expressed CD44v9 at higher levels, the population of CD44v9-positive cells was not decreased, although CD44v9 expression levels were markedly downregulated (Supplementary Fig. 2A). Our gastric cancer PDCs express wild-type *KRAS*, while *KRAS*-mutated cancer cells are known to be resistant to EGFR inhibitors.^21^ To test whether the EGFR inhibitors still affect CD44v-positive cells in *KRAS*-mutated cancers, we additionally examined the effect of EGFR inhibitors in SW480, a *KRAS*-mutant colorectal cancer cell line that is resistant to EGFR inhibition, as determined by the DepMap (https://depmap.org/portal/).^22^ We found that EGFR inhibition by afatinib led to a marked decrease in the population of CD44v9-expressed cells in SW480 cells (Supplementary Fig. 2B, C). These observations indicate that EGFR inhibition could lead to a decrease in CD44v9-positive cell populations in gastric and colorectal cancers.

### EGFR maintains CD44v9-positive cell proliferation through AKT and ERK-dependent CD44v9 protein expression

**Fig. 4:**
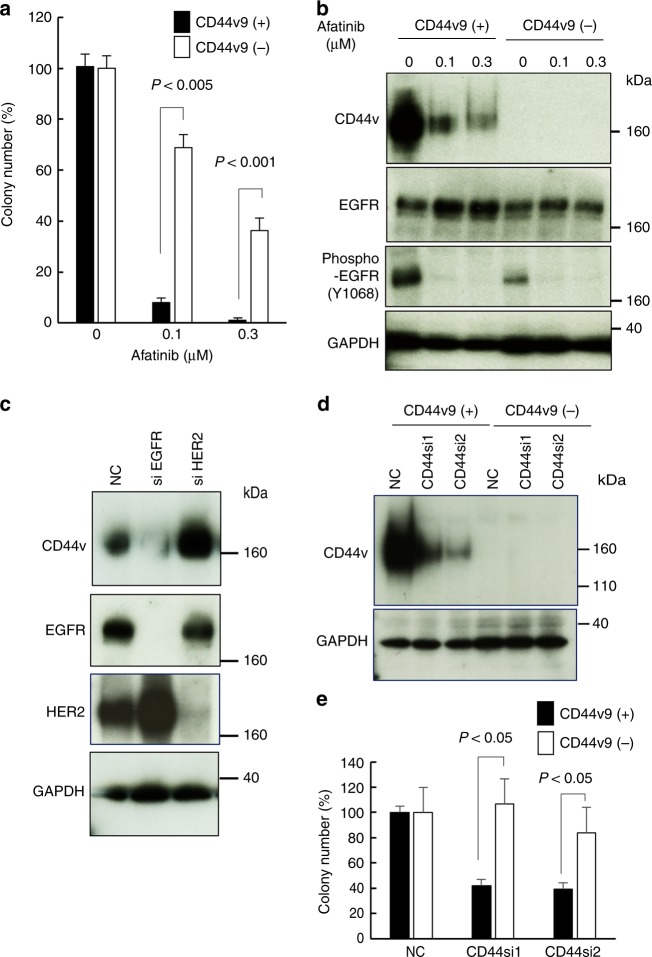
Afatinib causes CD44v downregulation leading to selective reduction of CD44v9-positive gastric cancer cells. **a** Preferential clonogenic growth inhibition of CD44v9-positive gastric cancer cells by afatinib. CD44v9-positive and CD44v9-negative cells sorted from JSC15-3 cells were treated with afatinib at the indicated concentrations for 14 days. Colony numbers were evaluated as described in the Materials and Methods section. **b**, **c** EGFR-dependent CD44v expression in gastric cancer cells. In **b**, CD44v9-positive and CD44v9-negative cells sorted from JSC15-3 cells were treated with afatinib at the indicated concentrations for 6 days. CD44v expression as well as EGFR autophosphorylation at Tyr1068 and EGFR levels were examined by immunoblot analysis. In **c**, JSC17-7 cells were treated with *EGFR*- or *HER2*-targeting siRNA for 4 days. CD44v expression as well as EGFR and HER2 protein levels were examined by immunoblot analysis. **d**, **e** Involvement of CD44v proteins in clonogenic growth of CD44v9-positive gastric cancer cells. CD44v9-positive and CD44v9-negative cells sorted from JSC15-3 cells were treated with *CD44*-targeting siRNAs as well as control siRNA (NC). Forty-eight hours after the siRNA treatment, *CD44v* knockdown was confirmed by immunoblot analysis (**d**). At 14 days after the siRNA treatment, colony numbers were evaluated (**e**). In clonogenic growth assays, error bars indicate standard deviation. Statistical significance was evaluated by Student’s *t* test

To determine the precise effect of afatinib on the maintenance of CD44v9-positive gastric cancer cell populations, we examined the effect of afatinib on CD44v9-positive and CD44v9-negative cells. As shown in Fig. 4a, b, afatinib preferentially suppressed CD44v9-positive cell proliferation at the same dosage of EGFR phosphorylation inhibition. These observations indicated that afatinib could eradicate the CD44v9-positive cell population through selective growth perturbation. In the CD44v9-positive JSC15-3 cell population (Fig. 4b) and in JSC17-7 cells where CD44v proteins were ubiquitously expressed (Supplementary Fig 2), we observed marked attenuation of CD44v9 expression by afatinib. These observations suggest that EGFR-mediated signalling could be required for CD44v9 expression. To further confirm this, we knocked down *EGFR* in JSC17-7 cells. Because afatinib inhibits not only EGFR but also another family member, HER2/ErbB2/neu,^23^ we further compared the effect of *EGFR* knockdown with that of *HER2*. Downregulation of *EGFR* but not *HER2* led to CD44v protein downregulation (Fig. 4c). These observations indicate that EGFR but not HER2 would be required for CD44v protein expression. To confirm whether EGFR inhibition-mediated CD44v downregulation could cause the CD44v9-expressing cell-selective growth inhibition, we examined the effect of *CD44* knockdown on the proliferation of CD44v9-positive and CD44v9-negative cells sorted from the gastric cancer PDCs (Fig. 4d). We observed preferential growth inhibition of the CD44v9-positive cells by the siRNAs (Fig. 4e). Collectively, these results indicate that CD44v9 protein downregulation could be a critical event in the selective anti-proliferative effect of afatinib in CD44v9-positive cells.

**Fig. 5:**
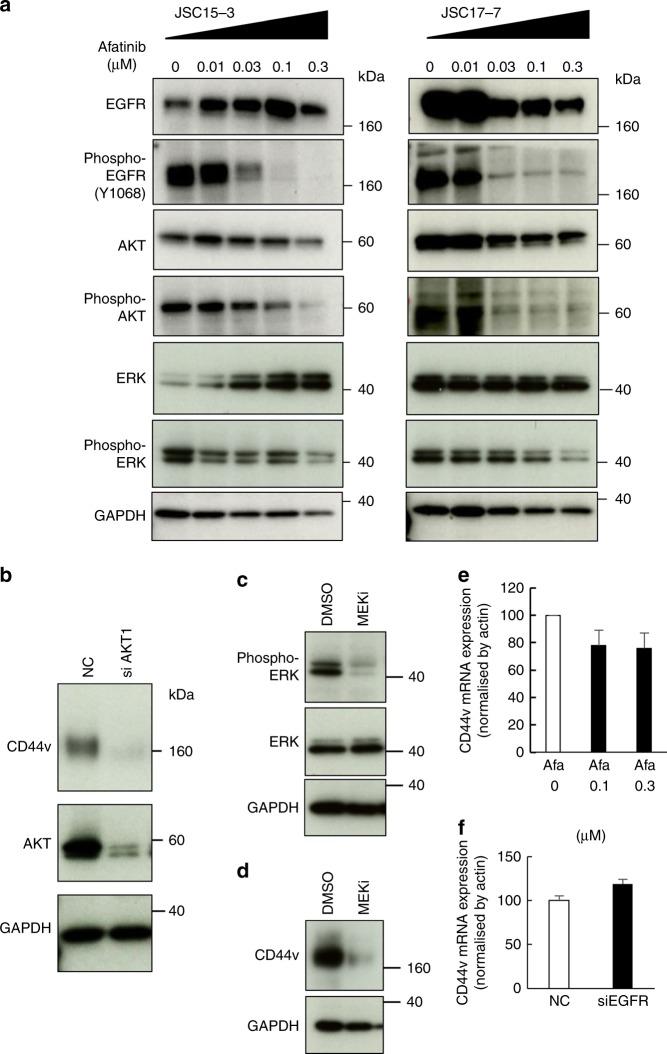
Requirement of the EGFR–AKT/ERK pathways for CD44v protein expression in gastric cancer cells. **a** AKT and ERK dephosphorylation in afatinib-treated gastric cancer cells. Cells were treated with afatinib at the indicated concentrations for 6 days. AKT, ERK and EGFR phosphorylation as well as their protein levels were examined by immunoblot analysis. **b** Requirement of AKT in CD44v protein expression in gastric cancer cells. JSC17-7 cells were treated with *AKT1*-targeting siRNA as well as control siRNA (NC). Seventy-two hours after the siRNA treatment, CD44v and AKT levels were examined by immunoblot analysis. **c**, **d** JSC17-7 cells were left untreated (DMSO) or treated with a MEK inhibitor (MEKi), trametinib, at 10 nM for 2 h (**c**) or for 6 days (**d**). ERK phosphorylation as well as its protein level (**c**) or CD44v protein expression (**d**) was examined by immunoblot analysis. **e**, **f** Effect of afatinib or EGFR inhibition on *CD44v* mRNA expression. The total RNA was extracted from JSC15-3 cells treated with afatinib at the indicated concentrations for 6 days (**e**) or JSC17-7 cells treated with *EGFR*-targeted siRNA for 4 days (**f**) and cDNA was synthesised. *CD44* expression was quantified by quantitative PCR. Expression was normalised against *ACTB*. Error bar indicates standard deviation

EGFR regulates its downstream protein kinase cascade.^24^ In the gastric cancer PDCs, afatinib decreased the phosphorylation of Ser473 in AKT and the phosphorylation of Thr292/Tyr204 in ERK, which are critical for their activation (Fig. 5a). Thus, we tested the effect of AKT inhibition on CD44v9 expression to determine whether AKT is involved in CD44v expression. As shown in Fig. 5b, *AKT* knockdown efficiently reduced CD44v expression in JSC17-7 cells. The ERK inhibition by trametinib, a MEK-specific inhibitor, also decreased CD44v expression (Fig. 5c, d). We additionally examined PTEN, PI3K and mTOR levels as well as the levels of p70S6K and 4E-BP1, downstream molecules of mTOR, after EGFR inhibitor treatment (Supplementary Fig. 6A). We did not observe marked change of these molecules after afatinib treatment, though 4E-BP1 and phospho-4E-BP1 levels were slightly decreased only in JSC15-3 cells. Moreover, CD44v expression was not affected by the treatment with temsirolimus, an mTOR inhibitor, either (Supplementary Fig. 6B, C). To further examine how EGFR regulates CD44v expression, we analysed CD44v expression at the transcriptional level. As shown in Fig. 5e, f, afatinib treatment or *EGFR* knockdown did not markedly downregulate *CD44v* mRNA expression. Moreover, afatinib did not alter *CD44v* splicing patterns either (Supplementary Fig. 7A). These observations suggest that EGFR regulates CD44v expression mainly at the translational level. Hence, we further examined CD44v protein stability before and after afatinib treatment by blocking protein synthesis with cycloheximide and subsequently chasing the CD44v protein levels. We did not observe significant differences in CD44v protein stability after afatinib treatment (Supplementary Fig. 7B).

Taken together, these observations suggest that EGFR inhibitor attenuates CD44v protein expression though an AKT-dependent and ERK-dependent pathway, which reduces the therapy-resistant gastric cancer subpopulation.

### Combined therapeutic effect of afatinib and cytotoxic antitumor agents on gastric cancer PDCs

**Fig. 6:**
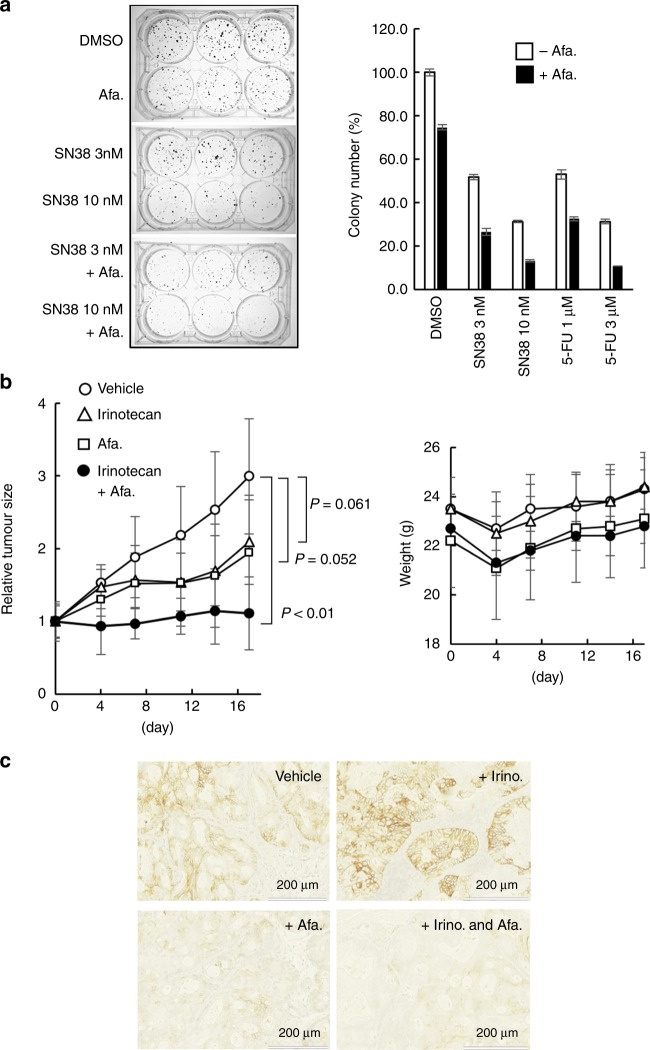
Therapeutic effect of EGFR inhibitor combined with cytotoxic antitumor agents in gastric cancer cells. **a** Co-treatment of afatinib potentiates the anti-proliferative effects of cytotoxic antitumor agents in gastric cancer cells. JSC15-3 cells were treated with 30 nM afatinib and SN-38 or 5-FU at the indicated concentrations for 14 days. Colony numbers were evaluated as described in the Materials and Methods section. Error bars indicate standard deviation. Left-hand images indicate the combinational effect of afatinib with SN-38. **b** In vivo tumour growth inhibition by co-treatment with afatinib and irinotecan. BALB/c-nu/nu mice were injected subcutaneously with JSC15-3 cells. Afatinib (30 mg/kg/day) and irinotecan (50 mg/kg/day) were administered intraperitoneally as described in the Materials and Methods section. Tumour growth (left panel) and body weight change of mice (right panel) during the treatment are shown. Error bars indicate standard deviation. Statistical significance was evaluated by Student’s *t* test. **c** CD44v9 expression in tumour tissues. Xenograft tumours in (**b**) were collected on day 19 and subjected to immunohistochemistry with anti-human CD44v9 antibody

To determine the effect of afatinib on the antitumor efficacy of cytotoxic antitumor agents, we treated JSC15-3 cells with afatinib and SN-38 or 5-FU. As shown in Fig. 6a, we observed a significance decrease in the number of persister cells after co-treatment of afatinib with SN-38 or 5-FU in the in vitro colony-formation analysis. Similarly, *EGFR* knockdown also enhanced the anti-proliferative effect of SN-38 (Supplementary Fig. 8A, B). We further tested the cooperative effect of afatinib and irinotecan in the mouse xenograft model (6 mice/group and 24 mice totally). Before treatment, each group of mice showed similar body weight (22–24 g) (Fig. 6b). Co-treatment with afatinib and irinotecan led to statistically significant tumour growth inhibition with less effect on mouse body weight (Fig. 6b), or no visible adverse event in mice. In the afatinib-treated group, we observed an efficient reduction in CD44v9 expression (Fig. 6c). These observations suggest that EGFR inhibition could be an effective strategy to eradicate any persister cells after chemotherapy to achieve a better treatment outcome.

## Discussion

Cancer stem cells with highly tumorigenic and drug-resistant potential have been identified in various cancer types.^25^ Cell subpopulations that express the cell surface CD44v9 protein are thought to be a gastric cancer stem cell fraction.^9–12,26^ However, various factors are related to the drug-resistant phenotype, and it has not been established as to what extent a cancer stem cell population could contribute to the early phases of drug resistance.^27^ In this study, we demonstrated that CD44v9-positive cells are enriched in residual cancer cells after SN-38 or 5-FU treatment in patient-derived gastric cancer cell models. We further showed that CD44v9 conferred resistance to SN-38. However, exogenous CD44v9 overexpression did not cause 5-FU resistance, although CD44v9-positive cells were also enriched among the residual cancer cells after 5-FU treatment. Our transcriptome and subsequent gene ontology analyses revealed that CD44v9-positive cells from JSC15-3 cells expressed several factors that were related to drug efflux or drug metabolism (unpublished observations). Therefore, these factors could be involved in the 5-FU-resistant phenotype exhibited by the cells.

We utilised our original antitumor compound-related gene expression database and identified EGFR inhibitors, such as afatinib and erlotinib, as agents that target drug-tolerant CD44v9-positive cells in gastric cancer. Several recent studies have also suggested the potential effects of EGFR inhibitors on cancer stem cells in other cancer types.^28,29^ However, these studies were performed based on treatment with much higher dosages of the inhibitors than the clinically applicable doses. In our present study, we showed that the EGFR inhibitors targeted CD44v9-positive cells at clinically relevant concentrations.^21^ Among the EGFR inhibitors, afatinib eliminated CD44v9-positive cell populations more strongly than erlotinib (Fig. 3c, d). We first speculated that the inhibitory effect of afatinib on another target, HER2, could cause the stronger activity of this kinase inhibitor. However, this was not the case, because knockdown of *HER2* did not affect CD44v9 levels. Another potential explanation would be the difference in the mechanisms of EGFR inhibition between the two agents; erlotinib reversibly inhibits EGFR, while afatinib is an irreversible kinase inhibitor.^30^ Further studies would clarify the differences between the EGFR inhibitors in their anti-proliferative effects on CD44v9-positive cell populations. EGFR and HER2 share similar downstream signalling, while our data indicate that EGFR could predominantly be involved in the regulation of CD44v expression (Fig. 4c). In our study, we mainly used gastric cancer PDC, JSC17-7 cells for the analysis. This PDC relatively overexpresses EGFR, but not HER2 (Supplementary Fig. 1B), and could be dependent more on EGFR than on HER2. On the other hand, some previous reports suggest that CD44 could interact not only with EGFR but also with HER2 in cancer cells.^31–33^ Considering these observations, we could speculate that the CD44v regulation by receptor tyrosine kinases could depend on cell types or cell context.

In the gastric PDCs, EGFR inhibitors mainly suppressed CD44v9 protein expression without affecting CD44v9 protein stability. These observations suggest that EGFR-dependent signalling could regulate CD44v9 protein translation. However, as a downstream signalling cascade of EGFR, our data suggest that the AKT signalling pathway could have a role in the regulation of CD44v9 levels. We also tested the effect of temsirolimus, an inhibitor of mTOR, which is a downstream factor of AKT and is involved in protein translation, on CD44v levels. Nonetheless, temsirolimus treatment did not affect CD44v9 levels in our gastric cancer PDCs (Supplementary Fig. 6B, C). Conversely, previous reports have shown that EGFR directly interacts with CD44,^32,33^ although we could not reproduce this interaction in our gastric cancer PDCs (unpublished observations). Further analysis would elucidate the signalling pathways involved in EGFR-dependent CD44v protein regulation.

In previous clinical trials of gastric cancer, some EGFR-targeting agents, such as panitumumab, cetuximab and nimotuzumab, have been tested without success.^34^ These studies were basically undertaken in unselected gastric cancer patients or in patients with EGFR-overexpressing gastric tumours. In our tissue microarray analysis, we did not find any significant correlation between CD44v9 expression and EGFR/phospho-EGFR expression (unpublished observations). These data suggest that the effect of EGFR inhibitors on gastric tumours would not necessarily be dependent on *EGFR* amplification, but could rely on other factors such as the presence of CD44v9-positive cell populations in the tumour tissues. In this respect, further retrospective subclass analyses of the previous clinical trials could validate the relationship of the effectiveness of EGFR inhibitors with CD44v9 expression in clinical settings. To translate our findings into clinical trials, high CD44v expression in gastric cancer tissues could be utilised as criteria to select patients to be enrolled in the EGFR-targeted therapy in combination with the cytotoxic antitumor agent. Because the EGFR inhibitor suppressed CD44v expression even in *KRAS*-mutated cancer cells, the EGFR inhibitors could also be applicable to the *KRAS*-mutated tumours to target the drug-tolerant CD44v-positive cancer cells.

In summary, by utilising our in silico drug screening approaches, we identified EGFR inhibitors, such as afatinib, as potential agents to target pre-existing drug-tolerant gastric cancer cells. Development of new agents for new disease-related phenotypes is a time-consuming and expensive work including multi-step in vivo validation studies. In silico drug screening is a time-saving and cost-saving strategy, and could be a suitable method with which to gain insights into new pharmacological treatment strategies through a drug repositioning approach.

## Supplementary information

Supplementary File

## Data Availability

The gene expression data have been deposited in Gene Expression Omnibus (GEO) and are accessible through the accession number GSE129747. The data will be released on March 31, 2020. Other datasets obtained in this study or materials are available from the corresponding authors on reasonable request.
