# Supplementary material for: In silico chemical screening identifies epidermal growth factor receptor as a therapeutic target of drug-tolerant CD44v9-positive gastric cancer cells
Source: Br J Cancer. 2019 Oct 14;121(10):846–56. doi: 10.1038/s41416-019-0600-9 (PMC6889183; doi:10.1038/s41416-019-0600-9)
Supplement: Supplementary file 1 — Supplementary File [file 41416_2019_600_MOESM1_ESM.pdf]

Supplementary Materials and Methods

Cell lines and cell culture conditions

Among the gastric cancer patient-derived cells (PDCs), JSC15-3 cells were cultured in PCM-2 medium and 100 μg/mL kanamycin. JSC17-2 cells were maintained in ACL-4/F-12 (1:1) medium supplemented with 5% heat-inactivated fetal bovine serum (FBS) and 2 μg/mL kanamycin. JSC17-7 cells were maintained in ACL-4/RPMI (1:1) medium supplemented with 5% heat-inactivated FBS and 2 μg/mL kanamycin. We obtained human gastric cancer MKN45 and MKN7 cells as described previously[35], human gastric cancer Kato III cells from the JCRB cell bank (Osaka, Japan), and human colorectal cancer SW480 cells from American Type Culture Collection, where the cell lines were authenticated by short tandem repeat analysis. KATO III, SW480, MKN45 and MKN7 cells were cultured in RPMI 1640 medium supplemented with 10% heat-inactivated FBS and 2 μg/mL kanamycin. JSC15-3 cells were cultured in collagen-coated dishes while other cell lines were maintained in normal dishes. To evaluate spheroid growth, cells were cultured in 96-well EZ-BindShut II plate (3-D

culture, Iwaki) and cell viability was evaluated using CellTiter-Glo luminescent cell viability assay (Promega).

Sequencing of *KRAS* gene

Total RNA was extracted and cDNA was synthesized as described in the RT-qPCR section. Protein coding region of *KRAS* gene was amplified by PCR from cDNA using the KOD Plus Neo kit (Toyobo Co., Ltd., Osaka, Japan) and was sequenced with 3130 genetic analyzer (Applied Biosystems).

Vector construction

Full-length cDNA for *CD44v* was amplified by PCR using cDNA extracted from human patient-derived gastric cancer JSC15-3 cells as a template and was sequenced as described previously[19]. The primers used were as follows: *CD44* (full) forward primer: 5'-TTTCTCGAGCGGACACCATGGACAAGTTTTG-3', *CD44* (full) reverse primer: 5'-AAAATCGATTAATGGTGTAGGTGTTACACCC-3'. The amplified cDNA was

CD44v9, which was cloned into a pLNCX2 retrovirus vector (Takara Bio, Shiga, Japan) to generate pLNCX2-CD44v9.

Antibodies for western blot analysis

We used the following antibodies for western blot analysis: anti-human CD44 (1:400; Cell Signaling Technology), anti-human EGFR (1:1000; Cell Signaling Technology), anti-human phospho-EGFR (Y1068) (1:1000; Cell Signaling Technology), anti-human HER2 (1:1000; Cell Signaling Technology), anti-human AKT (1:500; Cell Signaling Technology), anti-human phospho-AKT (S473) (1:500; Cell Signaling Technology), anti-human phospho-ERK (T292/Y204) (1:1000; Cell Signaling Technology), anti-human ERK (1:1000; Cell Signaling Technology), anti-human PTEN (1:1,000; Cell Signaling Technology), anti-human phosphatidylinositol-3 kinase (PI3K) p110 subunit (1:500; Cell Signaling Technology), anti-human PI3K p110 subunit (1:500; Cell Signaling Technology), anti-human PI3K p85 subunit (1:500; Cell Signaling Technology), anti-human mTOR (1:1,000; Cell Signaling Technology), anti- anti-human phospho-p70S6 kinase (p70S6K) (T389) (1:500; Cell Signaling Technology), anti-human p70S6K (1:500; Cell Signaling Technology), anti-human phospho-4E-BP1 (S65) (1:500; Cell Signaling Technology),

anti-human 4E-BP1 (1:500; Cell Signaling Technology), anti-human FGFR2 (1:500; Cell Signaling Technology), anti-human MET (1:500; Cell Signaling Technology) and anti-human GAPDH (1:2000; Santa Cruz Biotechnology, Dallas, TX).

siRNA

siRNAs used in this study were as follows:

siRNA against *EGFR* (5'-CCAUAAAUGCUACGAAUAUtt-3'), siRNA against *HER2* (5'-GCUCAUCGCUCACAACCAAtt-3'), siRNA against *AKT1* (5'-GCGUGACCAUGAACGAGUUtt-3'), siRNA against *CD44* (#1) (5'-GCTGACCTCTGCAAGGCTTTCAATA-3'), siRNA against *CD44* (#1) (5'-GCAAGTCTCAGGAAATGGTGCATTT-3').

Reverse transcription-PCR (RT-PCR)

Total RNA was extracted, and cDNA was synthesized as described in the Materials and Methods. Each variant exon-containing *CD44* DNA fragment was amplified by PCR with the primer sequences shown below and subjected to agarose gel electrophoresis.

CD44 forward primer for variant exon3: 5'- TGGGAGCCAAATGAAGAAAA -3', forward primer for variant exon 4: 5'- TCAACCACACCACGGGCTTT -3', forward primer for variant exon 5: 5'- GTAGACAGAAATGGCACCAC -3', forward primer for variant exon 6: 5'- GAGGCAACTCCTAGTAGTAC -3', forward primer for variant exon 7: 5'- CAGCCTCAGCTCATACCAGC -3', forward primer for variant exon 8: 5'- TCCAGTCATAGTACAACGCT- -3', forward primer for variant exon 9: 5'- CAGAGCTTCTCTACAATCACA -3', forward primer for variant exon 10: 5'- GGTGGAAGAAGAGACCCAAA -3'; *CD44* reverse primer: 5'-TTTGCTCCACCTTCTTGACTCC-3'.

Primers to quantitate the expression of stem cell- and differentiation-related factors in RT-qPCR

Primer sequences for RT-qPCR were as follows:

MUC5AC forward primer: 5'-CTCTGTGGGGACTTCAACG-3', *MUC5AC* reverse primer: 5'-ATTCCATGGGTGTCAGCTTG-3',

MUC6 forward primer: 5'-AGGCAGCAACATCGAAGG-3', *MUC6* reverse primer: 5'-TCCTCGTGGTCGAAGTACTCA-3',

MUC2 forward primer: 5'- CCTCATGAATCATTGCTCCG-3', *MUC2* reverse primer: 5'-TTTCTCCTCTTTGCAGCAGG-3',

SOX2 forward primer: 5'-GCTTAGCCTCGTCGATGAAC-3', *SOX2* reverse primer: 5'-AACCCCAAGATGCACAACTC-3',

ABCG2 forward primer: 5'- TTCCACGATATGGATTTACGG-3', *ABCG2* reverse primer: 5'-GTTTCCTGTTGCATTGAGTCC-3',

CD24 forward primer: 5'- CTGCTGGCACTGCTCCTA-3', *CD24* reverse primer: 5'-GTTGGATTTGGGGCCAACC-3'.

Experimental conditions and procedures of mouse xenograft study

Study design

Experimental groups: Upon subcutaneous injection of human gastric cancer cells, mice were divided into 4 groups (six mice per group) and treated with vehicle, irinotecan, afatinib, or irinotecan + afatinib.

Experimental unit: 6-week-old BALB/c-nu/nu mice (female).

Experimental procedures

Injection of human gastric cancer cells: JSC15-3 cells (2×10^6 cells/mouse) were suspended in 50 μL Hanks' balanced salt solution (HBSS) with 50 μL Matrigel (Corning) and were implanted subcutaneously in the right flanks of 6-week-old BALB/c-nu/nu mice.

Treatment: Therapeutic experiments (six mice per group) were started approximately 20 days after implantation when tumors reached 150–250 mm^3, as measured with calipers (day 0). Irinotecan (50 mg/kg/day) was prepared in PBS and administered i.p. on days 0, 6, and 13. Afatinib (30 mg/kg/day) was administered i.p. in 200 μL of solvent (Cremophor EL/ethanol/HCO40 (2:1:1)) on days 0, 4, 7, 11, 14, and 18. Control mice received the same volume of PBS on the day of irinotecan treatment and solvent on the day of afatinib treatment.

Weighting: Body weight and tumor size were measured during and after treatment every 3-4 days. The length (L) and width (W) of the tumor mass was measured, and the tumor volume (TV) was calculated as: $TV = (L\times W^2)/2$.

Euthanasia: At the end of the experiments, mice were euthanized by cervical dislocation.

Experimental animals

6-week-old BALB/c-nu/nu mice (female) (Charles River Laboratories, Japan), weight 15-25g.

Housing and husbandry

Animal facility: Standard animal experiment room at JFCR with automatic system of temperature, humidity and light regulation (temperature: 25 + 1°C; light/dark cycle: 12/12h; humidity: 50 + 10%).

Diet: Access to food [sterilized normal diet, CE-2 (CLEA Japan, Inc., Japan)] and sterilized water.

Cage: Sterilized plastic cages.

Cage companions: 3 animals/cage.

Bedding materials: high adsorbing bedding materials without dust. Changed every week. Environmental enrichment was done with sterile materials.

Sample size

6 mice/group (24 mice totally). We determined the sample size based on our previously-performed successful *in vivo* studies[19,35].

Allocating animals to experimental groups

Mice were divided into the above-mentioned 4 groups after randomization.

Experimental outcomes

1. To determine whether afatinib treatment could enhance the antitumor efficacy of cytotoxic antitumor agent, irinotecan, *in vivo*.

2. To examine whether afatinib treatment would decrease the CD44v9 expression in gastric cancer xenograft model.

Statistical methods

Statistical analysis was performed using Student's t-test to evaluate differences in the proliferation rates between treated and control groups.

Supplementary Reference

35 Mashima T, Oh-hara T, Sato S, Mochizuki M, Sugimoto Y, Yamazaki K, *et al.* p53-defective tumors with a functional apoptosome-mediated pathway: a new therapeutic target. *J Natl Cancer Inst.* 2005, **97**, 765-777.

Supplementary Figure Legends

Supplementary Figure 1. Expression of differentiation-related markers and oncogenic receptor tyrosine kinases (RTKs) in gastric cancer patient-derived cells (PDCs). (A) Expression of MUC2, MUC5AC and MUC6 in gastric cancer PDCs were determined by reverse transcription-quantitative PCR analysis. **(B)** Expression status of oncogenic RTKs that are frequently overexpressed in gastric cancer was examined in gastric cancer PDCs by immunoblot analysis.

Supplementary Figure 2. Alteration of CD44v9-positive cancer cell populations by afatinib in gastric and colorectal cancer cells. (A) JSC17-7 cells were left untreated (DMSO) or treated with afatinib at the indicated concentrations for 6 days. CD44v9-positive cancer cell populations in untreated control cells (black) or in drug-treated cells (red) were evaluated by flow cytometry. **(B),(C)** Colorectal cancer SW480 cells were left untreated (DMSO) or treated with afatinib at the indicated concentrations for 6 days. CD44v9-positive cell populations were evaluated by flow cytometry (B). EGFR autophosphorylation at Tyr1068 and EGFR protein levels were examined by immunoblot analysis (C).

Supplementary Figure 3. Drug resistance of CD44v9-expressing residual cancer cells after antitumor agent treatment and isolation of the *CD44v9* gene in gastric cancer gastric cancer patient-derived cells (PDCs). (A) Resistance of residual cancer cells to SN-38 after SN-38 and 5-FU treatment. JSC15-3 cells were left untreated (DMSO) or treated with 30 nM SN-38 or 3 μM 5-FU for 6 days. After the treatment, drug sensitivity of the residual cancer cells was examined by treating the cells with SN-38 at the indicated concentrations for 6 days. Error bars indicate standard deviation. **(B)** Evaluation of *CD44* variant forms expressed in JSC15-3 cells. Total RNA was extracted from JSC15-3 cells and cDNA was synthesized. Full-length *CD44v* cDNAs were amplified and subjected to agarose gel electrophoresis. The DNA was subsequently purified, and sequence analysis revealed that the amplified *CD44v* cDNA was the *CD44v9* form.

Supplementary Figure 4. Effect of antitumor agent treatment on *CD44v* splicing in gastric cancer PDCs. JSC15-3 cells were left untreated (DMSO) or treated with 30 nM SN-38 (A) or with 3 μM 5-FU (B) for 6 days. After the treatment, total RNA was extracted, and cDNA was synthesized. Each exon-specific *CD44v* fragment was amplified by polymerase chain reaction (PCR) and subjected to agarose gel electrophoresis.

Supplementary Figure 5. Cancer stem cell property of CD44v9-positive cells sorted from gastric cancer PDCs. (A) Sorting of CD44v9-positive and negative cells from JSC15-3 cells. CD44v9-positive and -negative cell populations were separated from JSC15-3 cells as described in the Materials and Methods. After cell sorting, we confirmed that more than 85% of each population was purified. **(B)** Expression of cancer stem cell markers in the CD44v9-positive and negative cells were determined by reverse transcription-quantitative PCR analysis. **(C)** Spheroid growth potential was exmined in the CD44v9-positive and negative cells as described in the Supplementary Materials and Methods.

Supplementary Figure 6. Alteration of PTEN/Phosphoinositide 3-kinase and mTOR pathways in afatinib-treated gastric cancer cells and effect of mTOR inhibitor. (A) Cells were treated with afatinib at the indicated concentrations for 6 days. PTEN/Phosphoinositide 3-kinase and mTOR pathway-related protein levels were examined by immunoblot analysis. **(B), (C)** JSC17-7 cells were left untreated (DMSO) or treated with an mTOR inhibitor, temsirolimus (Tem), at the indicated concentrations for two hours (B) or for 6 days (C). 4E-BP1 phosphorylation as well as its protein level (B) or CD44v protein expression (C) was examined by immunoblot analysis.

Supplementary Figure 7. Effect of afatinib treatment on CD44v splicing and protein stability in gastric cancer PDCs. (A) Effect of afatinib treatment on *CD44v* splicing. JSC15-3 cells were left untreated (DMSO) or treated with 0.3 μM afatinib for 6 days. After treatment, the expression of each variant exon-containing *CD44v* was examined as described in Supplementary Figure 4. **(B)** Effect of afatinib treatment on CD44v protein stability. JSC17-7 cells left untreated (DMSO) or pre-treated with 0.3 μM afatinib for 6 days were treated with 100 μg/mL cycloheximide (CHX) to attenuate

new protein synthesis for the indicated time periods. After treatment, cells were harvested and CD44v protein levels normalized against GAPDH levels were evaluated by immunoblot analysis and subsequent quantification of signals by Image J software.

Supplementary Figure 8. *EGFR* knockdown potentiates the anti-proliferative effect of SN38 in gastric cancer PDCs. (A) JSC15-3 cells were treated with *EGFR* siRNA or control siRNA (NC). Seventy-two hours after the siRNA treatment, EGFR protein levels were examined by immunoblot analysis. **(B)** Twenty-four hours after the siRNA treatment, the cells were seeded into 6-well plates and cultured for 14 days in the presence of DMSO (control) or 3 nM SN-38. Colony numbers were evaluated as described in the Materials and Methods. Error bars indicate standard deviation.

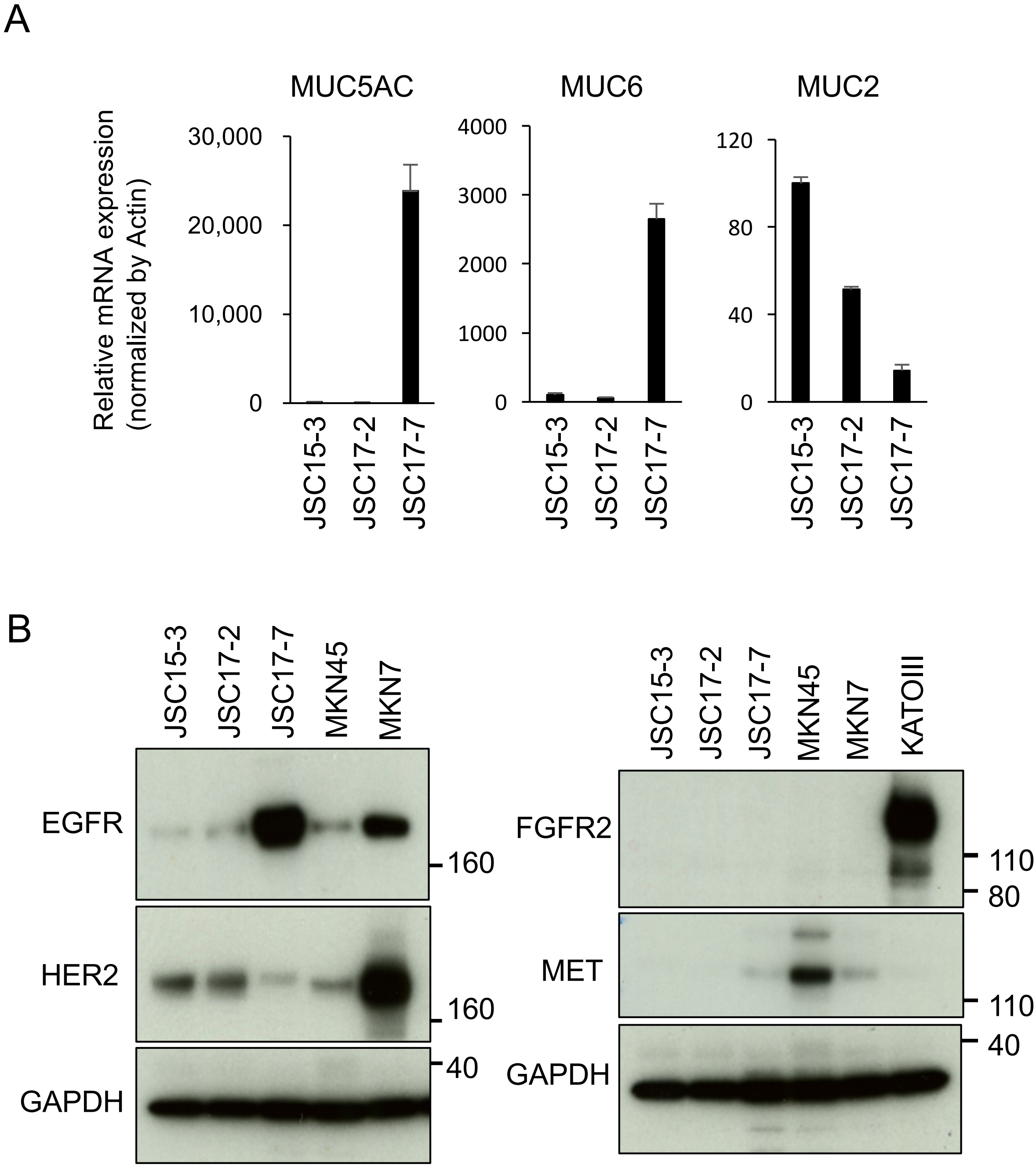

Mashima et al. Suppl. Fig. 1

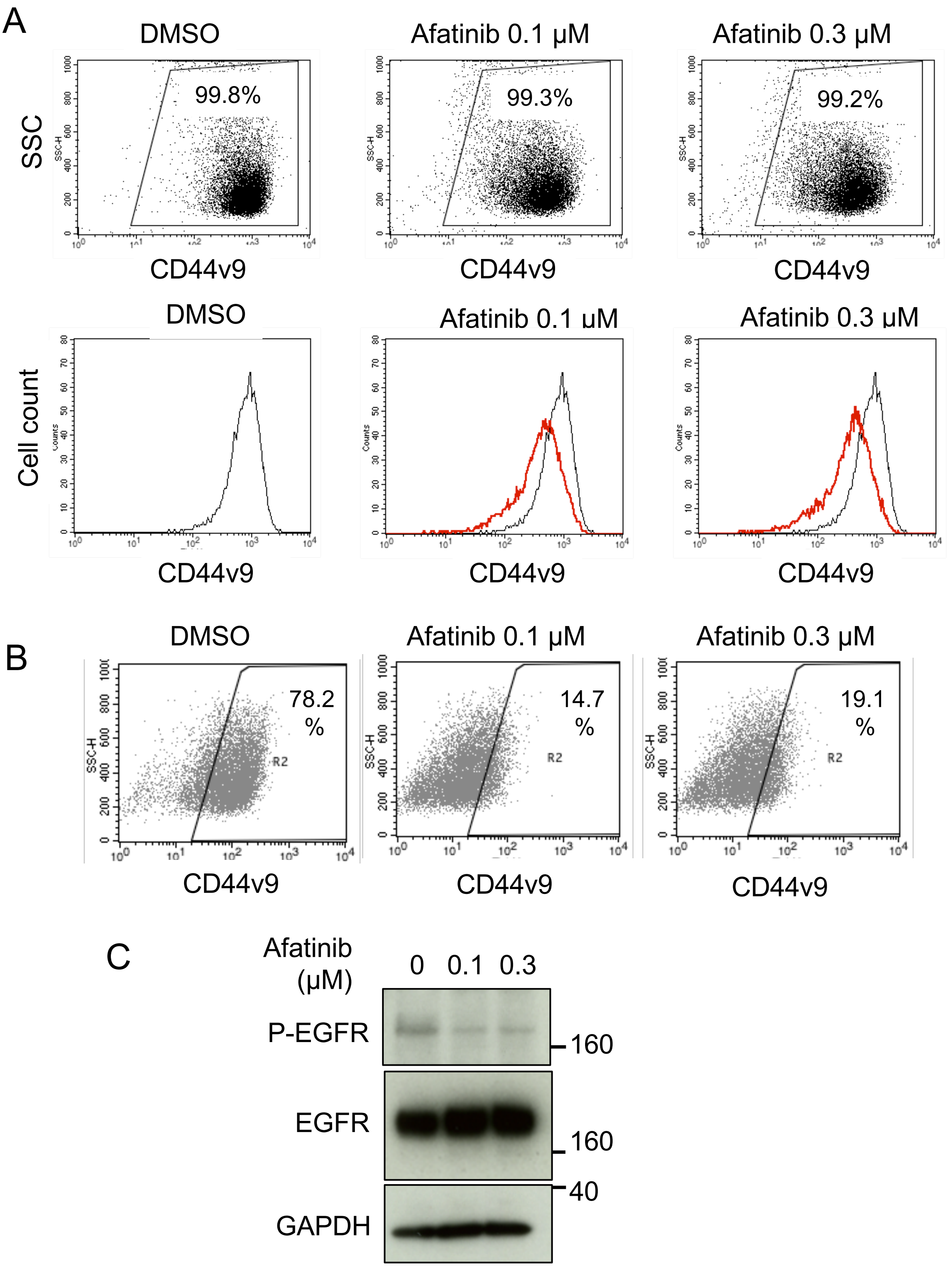

Mashima et al. Suppl. Fig. 2

A

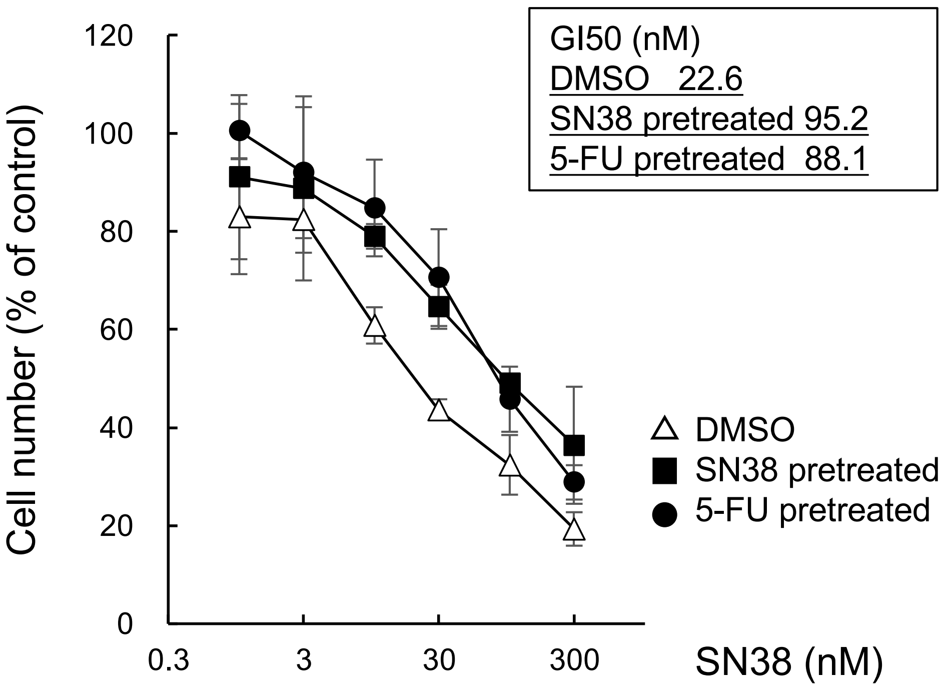

B

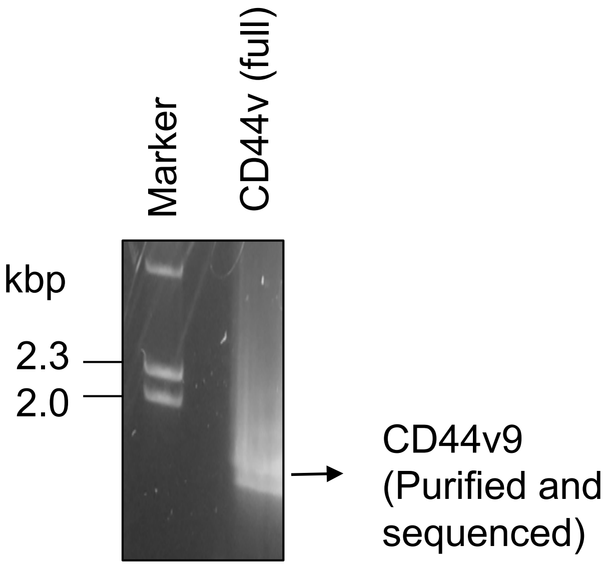

Mashima et al. Suppl. Fig. 3

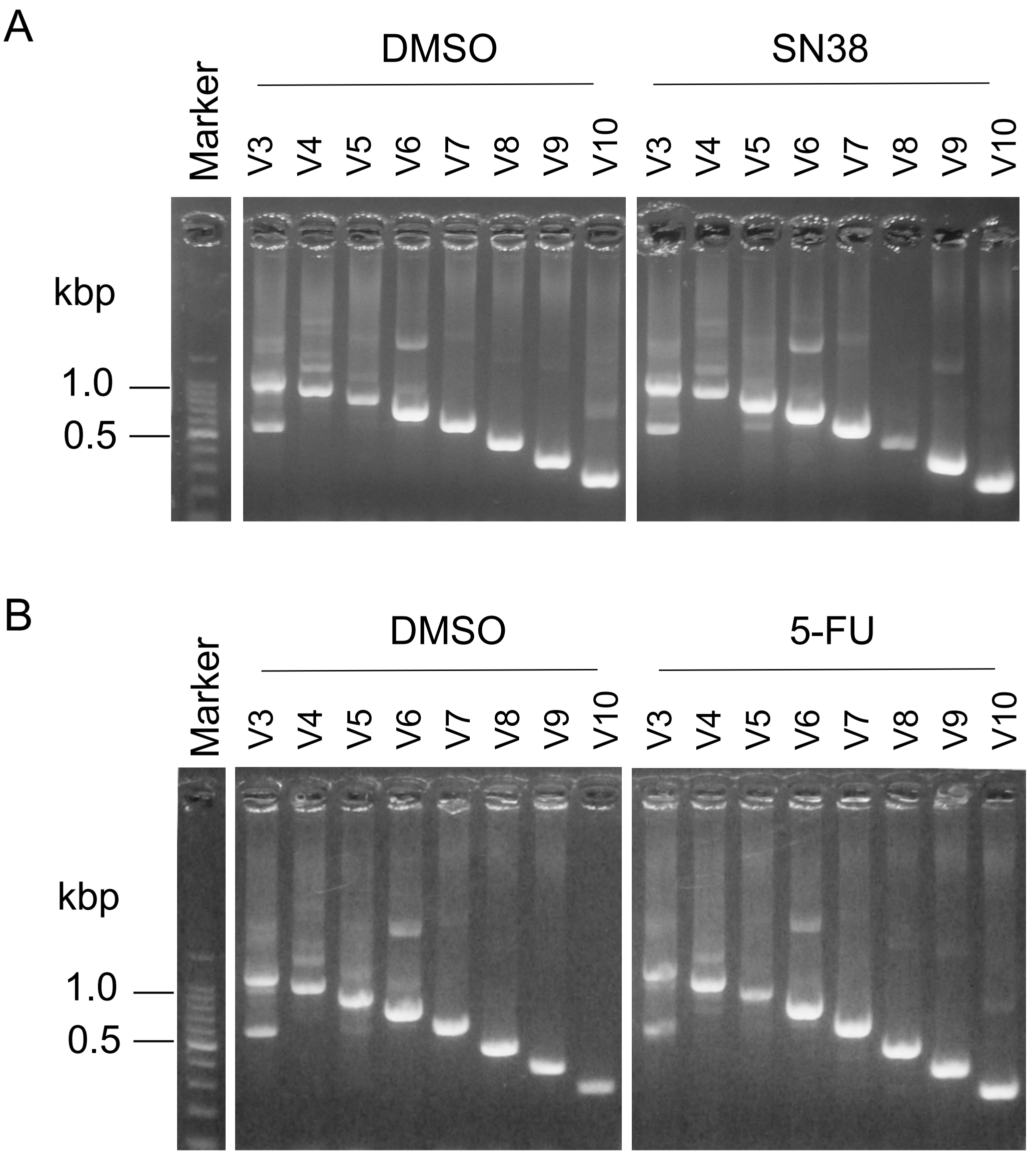

Mashima et al. Suppl. Fig. 4

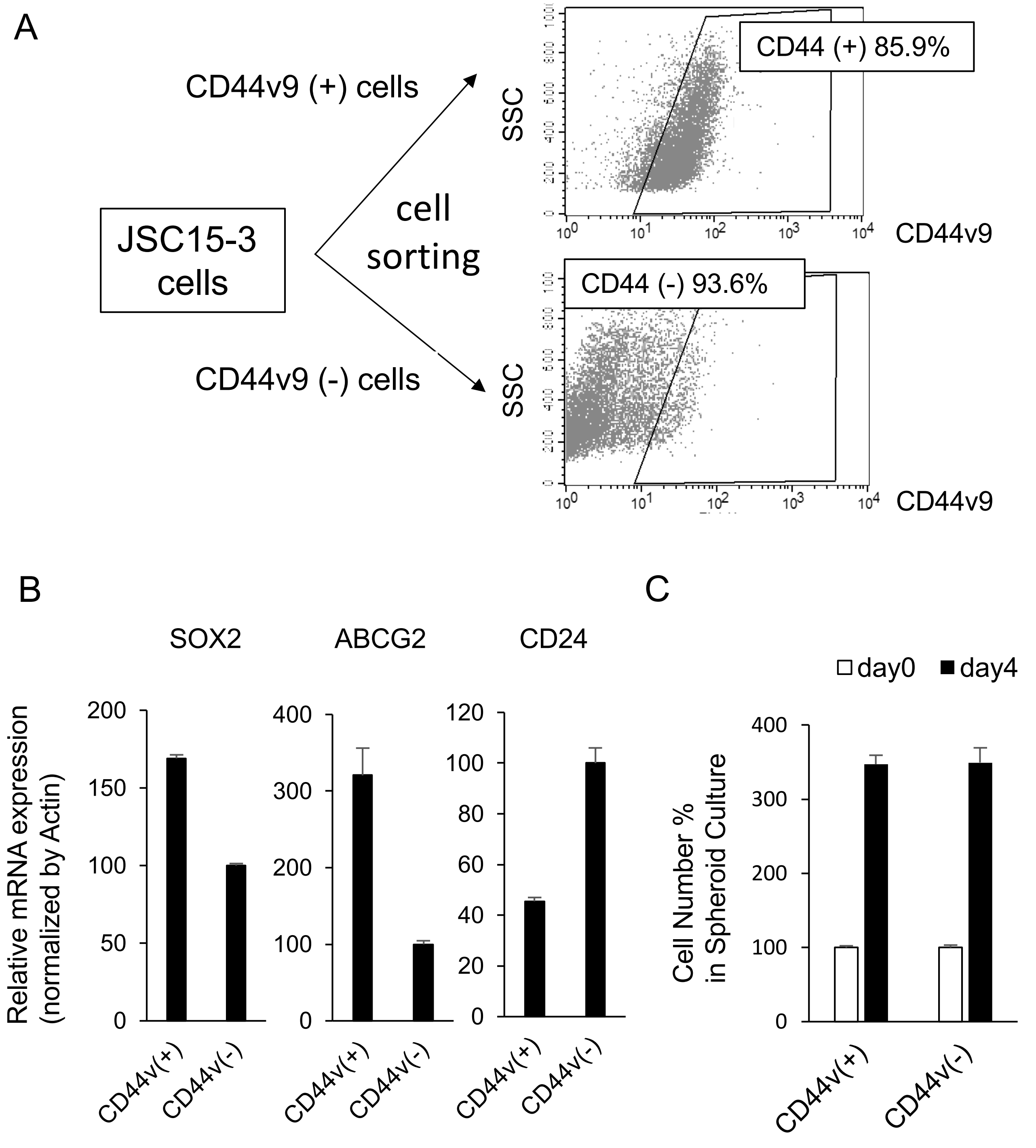

Mashima et al. Suppl. Fig. 5

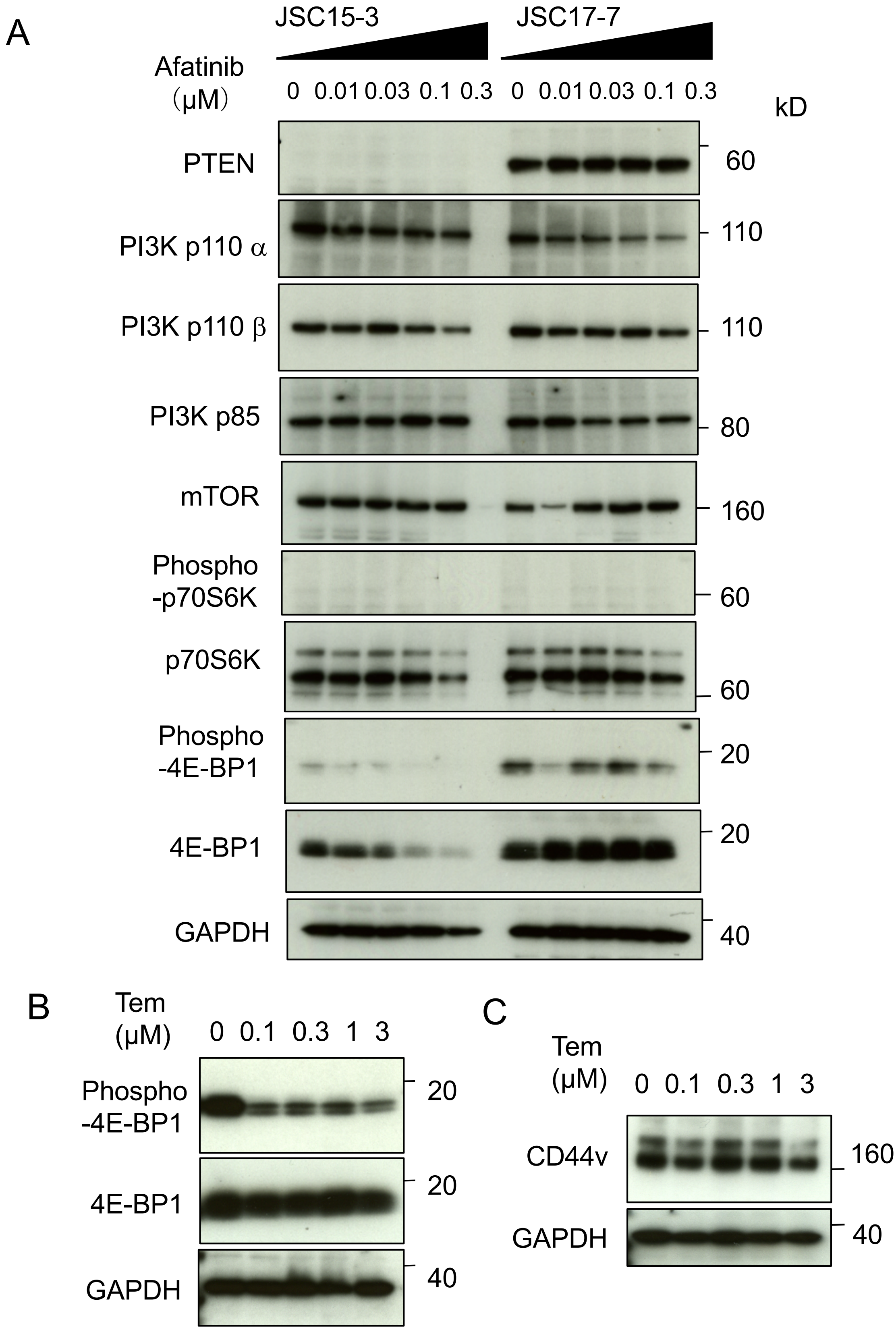

Mashima et al. Suppl. Fig. 6

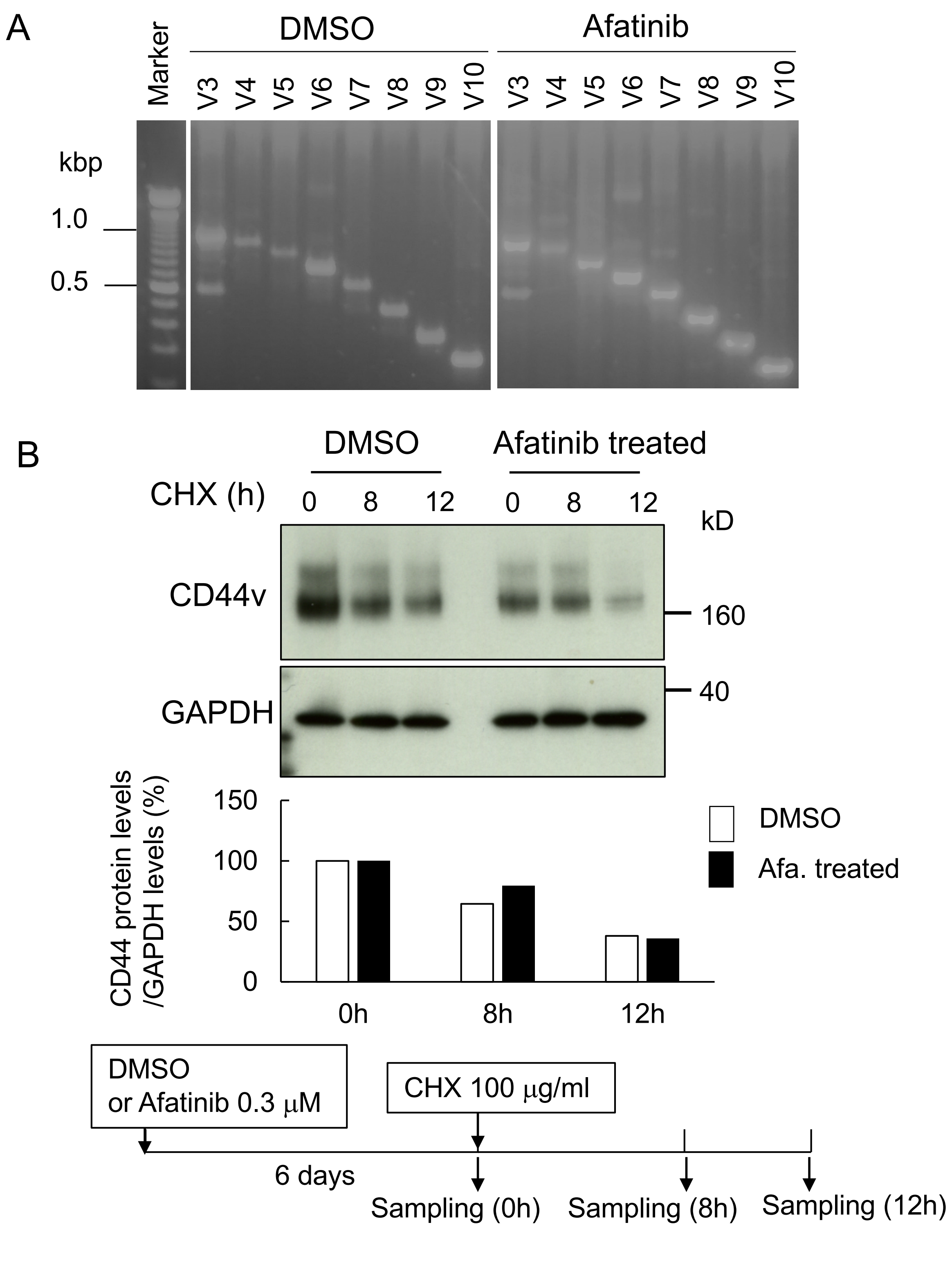

Mashima et al. Suppl. Fig. 7

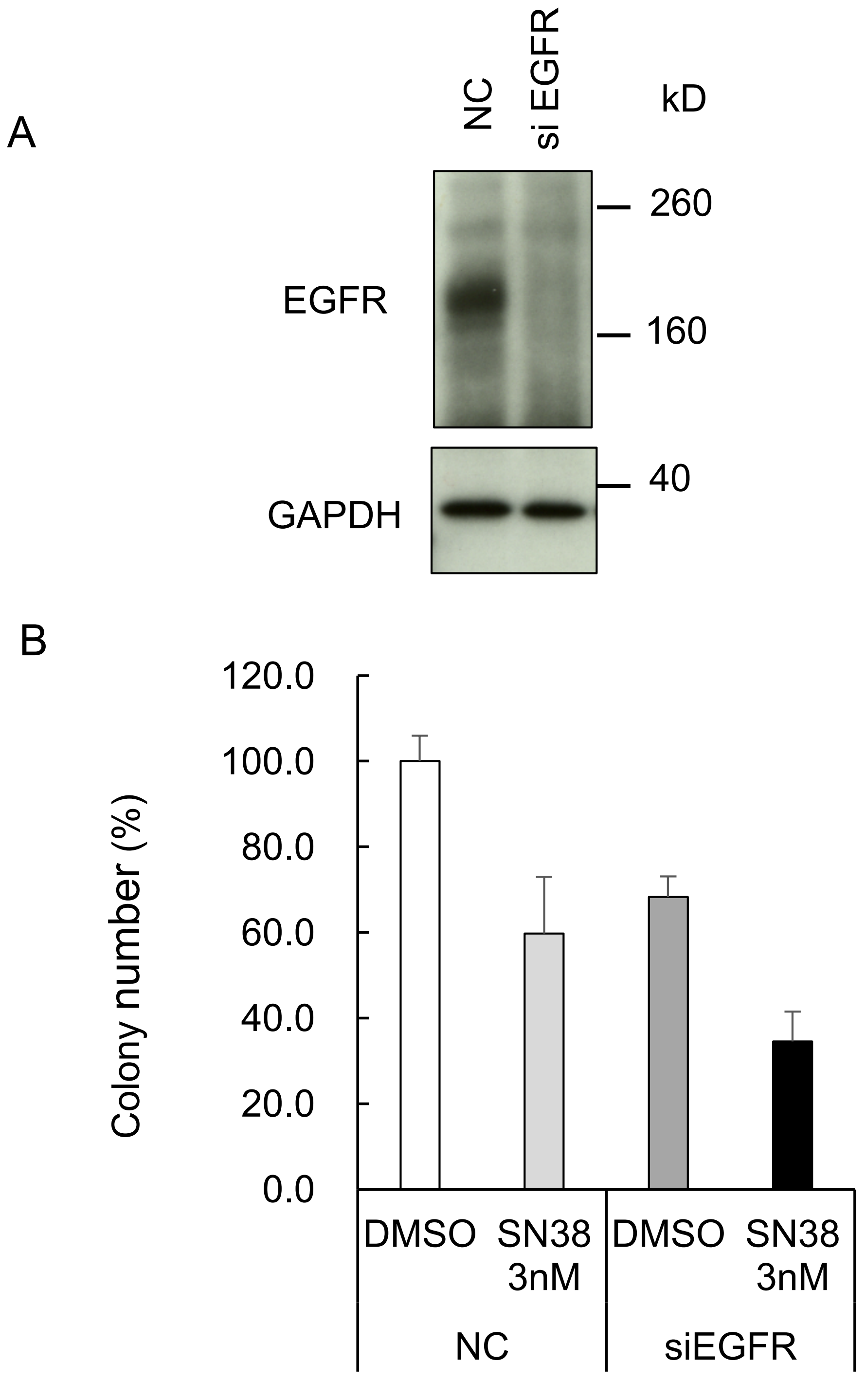

Mashima et al. Suppl. Fig. 8

Supplementary Table 1. Clinical information and CD44v9 expression in gastric tumor tissues by tissue microarray analysis.

No.	Age	Sex	Pathology diagnosis	TNM	Grade	Stage	CD44v expression score (IHC)
1	69	F	Adenocarcinoma	T1N0M0	1	Ia	0
2	53	M	Adenocarcinoma	T3N0M0	1	II	0
3	64	M	Adenocarcinoma	T3N0M0	1	II	0
4	62	M	Adenocarcinoma	T2N0M0	1	Ib	0
5	63	M	Adenocarcinoma	T2N0M0	1	Ib	0
6	51	M	Adenocarcinoma	T2N0M0	1	Ib	0
7	52	F	Adenocarcinoma	T3N0M0	1	II	1
8	63	M	Adenocarcinoma	T3N0M0	1	II	0
9	68	M	Adenocarcinoma	T2N0M0	1	Ib	0
10	44	M	Adenocarcinoma	T3N0M0	1	II	1
11	59	M	Adenocarcinoma	T2N0M0	1	Ib	2
12	65	M	Adenocarcinoma	T2N0M0	1	Ib	0
13	53	M	Adenocarcinoma	T2N0M0	1	Ib	0
14	54	F	Adenocarcinoma	T3N0M0	1	II	0
15	61	M	Adenocarcinoma	T3N0M0	1	II	0
16	60	F	Adenocarcinoma	T3N0M0	1	II	0
17	63	F	Adenocarcinoma	T3N0M0	2	II	0
18	58	M	Adenocarcinoma	T3N0M0	2	II	2
19	64	M	Adenocarcinoma	T2N0M0	2	Ib	0
20	75	M	Adenocarcinoma	T3N1M0	2	IIIa	0
21	44	M	Adenocarcinoma	T3N0M0	2	II	**1**
22	61	M	Adenocarcinoma	T2N0M0	2	Ib	0
23	61	M	Adenocarcinoma	T2N0M0	2	Ib	1
24	51	F	Adenocarcinoma	T3N0M0	2	II	0
2	64	M	Adenocarcinoma	T3N0M0	2	II	2
26	69	M	Adenocarcinoma	T2N0M0	2	Ib	0
27	46	F	Adenocarcinoma	T3N0M0	2	II	0
28	57	M	Adenocarcinoma	T3N0M0	2	II	1
29	60	M	Adenocarcinoma	T3N0M0	2	II	0
30	62	M	Adenocarcinoma	T2N0M0	2	Ib	0
31	65	M	Adenocarcinoma	T2N0M0	2	Ib	0
32	67	M	Adenocarcinoma	T3N0M0	2	II	0
33	52	M	Adenocarcinoma	T3N0M0	2	II	0
34	59	M	Adenocarcinoma	T2N0M0	2	Ib	0
35	68	M	Adenocarcinoma	T2N0M0	2	Ib	0
36	70	M	Adenocarcinoma	T3N0M0	2	II	1
37	68	F	Adenocarcinoma	T3N0M0	3	II	0
38	56	M	Adenocarcinoma	T3N0M0	3	II	3
39	71	M	Adenocarcinoma	T3N0M0	3	II	0
40	64	M	Adenocarcinoma	T3N1M0	3	IIIa	0
41	58	M	Adenocarcinoma	T3N0M0	3	II	1
42	60	M	Adenocarcinoma	T3N1M0	3	IIIa	3
43	70	M	Adenocarcinoma	T2N0M0	3	Ib	2
44	56	M	Adenocarcinoma	T4N1M0	3	IV	2
45	56	M	Adenocarcinoma	T3N0M0	3	II	1
46	65	M	Adenocarcinoma	T3N0M0	3	II	0
47	52	F	Adenocarcinoma	T2N0M0	3	Ib	0
48	64	M	Adenocarcinoma	T3N0M0	3	II	0
49	44	M	Adenocarcinoma	T2N0M0	3	Ib	0
50	71	M	Adenocarcinoma	T2N0M0	3	Ib	3
51	32	F	Adenocarcinoma	T3N0M0	3	II	0
52	53	F	Adenocarcinoma	T3N0M0	3	II	2
53	62	M	Adenocarcinoma	T3N1M0	3	IIIa	0
54	49	M	Adenocarcinoma	T2N0M0	3	Ib	0
55	65	M	Adenocarcinoma	T2N0M0	3	Ib	3
56	65	F	Adenocarcinoma	T2N0M0	3	Ib	0
57	72	M	Adenocarcinoma	T2N0M0	3	Ib	0
58	66	M	Adenocarcinoma	T2N0M0	3	Ib	3
59	67	M	Adenocarcinoma	T3N1M0	3	IIIa	0
60	48	F	Adenocarcinoma	T2N1M0	3	II	1
61	56	M	Adenocarcinoma	T3N0M0	3	II	0
62	47	M	Adenocarcinoma	T3N0M0	3	II	0
63	56	F	Adenocarcinoma	T2N0M0	3	Ib	1
64	71	M	Adenocarcinoma	T3N0M0	3	II	2
65	59	M	Adenocarcinoma	T3N1M0	3	IIIa	0
66	70	M	Adenocarcinoma	T2N0M0	3	Ib	0

CD44v9 expression levels determined by immunohistochemistry with anti-human CD44v9 antibody were semi-quantitatively scored in four levels (0: non, 1: low, 2: medium, 3: high).

Supplementary Table 2. Relationship between tumor grade and CD44v9 expression in gastric tumor tissues.

		CD44v expression		
		low/non	medium	high
Grade	1	13	2	1
	2	13	4	2
	3	17	4	9

CD44v9 expression was determined by immunohistochemistry as in Supplementary Table 1 and its relationship with tumor grade was summarized.

Supplementary Table 3. Information on the original gastric tumors of the established patient-derived cancer cells.

Cells	Age	Sex	Gross Type	Histrogical Type
JSC15-3	73	M	type2	well/moderately Differentiated
JSC17-2	53	F	type3	poorly Differentiated
JSC17-7	67	M	type3	poorly Differentiated

Supplementary Table 4 . Connectivity scoring analysis of the signature genes of the drug-tolerant CD44v9-positive gastric cancer cells in the JFCR_LinCAGE database.

rank	compound name	connectivity score	up_score	down_score
1	Afatinib	1	0.32	-0.81
2	Erlotinib	0.983	0.33	-0.781
3	Etoposide	0.972	0.33	-0.769
4	Gefitinib	0.896	0.272	-0.741
5	Neocarzinostatin	0.891	0.357	-0.65
6	Trametinib	0.873	0.141	-0.846
7	Romidepsin	0.864	0.209	-0.767
8	Gefitinib30	0.844	0.223	-0.731
9	Erlotinib30	0.843	0.214	-0.738
10	Vemurafenib	0.821	0.166	-0.762

Compounds in the database that showed high similarity in their gene signatures to the given compounds were extracted as described previously (16). Top 10 hit compound data are shown.
